# Taxonomic Approach to the Tachinid Flies *Dinera carinifrons* (Fallén) (Diptera: Tachinidae) and *Dinera fuscata* Zhang and Shima using Molecular and Morphometric Data

**DOI:** 10.1673/031.013.13901

**Published:** 2013-11-30

**Authors:** Erikas Lutovinovas, Igor Malenovský, Andrea Tóthová, Joachim Ziegler, Jaromír Vaňhara

**Affiliations:** 1 Department of Botany and Zoology, Faculty of Science, Masaryk University, Kotlářská 2, CZ-611 37 Brno, Czech Republic; 2 Department of Entomology, Moravian Museum, Hviezdoslavova 29a, CZ-627 00 Brno, Czech Republic; 3 Museum für Naturkunde, Leibniz Institute for Research on Evolution and Biodiversity at the Humboldt University, Invalidenstrasse 43, 101 15 Berlin, Germany

**Keywords:** 12S and 16S rDNA, Bayesian inference, canonical discriminant analysis, character evaluation, lectotype designation, maximum likelihood, mitochondrial molecular markers, Palaearctic region, parasitoids, principal component analysis, species delimitation, taxonomy, traditional morphometrics

## Abstract

Molecular phylogenetic and traditional morphometric methods were applied to examine six Palaearctic taxa of the taxonomically difficult tachinid fly genus *Dinera* Robineau-Desvoidy (Diptera: Tachinidae), with particular reference to *D. carinifrons* (Fallén) and *D. fuscata* Zhang and Shima. Results of a phylogenetic analysis based on the mitochondrial markers 12S and 16S rDNA and multivariate statistical analyses of 19 morphometric characters were used to delimit both species. A lectotype was designated for *D. carinifrons* to stabilize the nomenclature in the group. *Dinera carinifrons* has a transpalaearctic distribution and is present in Central Europe, especially in high altitudes of the Alps. It differs from the similar and closely related *D. fuscata* in that it has a slightly larger body size, a dense greyish microtrichosity on the body, and different head proportions. *Dinera fuscata*, as delimited here, is widespread in the Palaearctic region, including Europe. Slight differences in both molecular and morphometric characters were found between western (Europe and Iran) and eastern (China and Japan) populations of *D. fuscata*, which are interpreted as an intraspecific variation. Differential diagnosis between *D. carinifrons* and *D. fuscata* is provided in the form of a revised portion of the determination key to the Palaearctic *Dinera* by Zhang and Shima (2006).

## Introduction

The Tachinidae are generally regarded as a relatively recently radiating group of parasitoids that may be one of the largest and ecologically most important families of Diptera in the world (Stireman et al. 2006; Pape and Thompson 2013). The taxonomy of the Tachinidae is complicated, and even in wellstudied areas such as Central Europe there are open questions necessitating revisions of doubtful taxa that may eventually result in the description of new species or in new synonymies being established (Tschorsnig and Herting 1994).

The tachinid genus *Dinera* Robineau- Desvoidy (Diptera: Tachinidae) is a representative of the subfamily Dexiinae, tribe Dexiini (Herting 1984). Based on morphological characters, it is closely related to *Billaea*, and differences between these two groups are not sharp (Zhang and Shima 2006). Zhang and Fu (2012) treated *Dinera* as differing from *Billaea* in that it has a narrow vertex in males, its fronto-orbital plate is bare or sparsely covered with minute setulae, its fore-tarsi are distinctly longer than head height, and its anterodorsal setae are irregular in length on the hind tibia. Twenty-eight presently known species treated in *Dinera* are mostly restricted to the Old World. The genus is apparently missing in the Neotropical and Australasian Regions (O'Hara 2012). Seven species of *Dinera* are known from the Afrotropical Region (Crosskey 1980), 11 from the Oriental Region (Crosskey 1976; Zhang and Shima 2006; Zhang and Fu 2012), 1 is of Holarctic distribution (O'Hara and Wood 2004) and 10 species are currently known from the Palaearctic Region (Zhang and Shima 2006; Cerretti 2010; Zhang and Fu 2012). The biology of most *Dinera* spp. is still unknown, but at least a few species from Europe and North America were reared as solitary parasitoids from beetle larvae dwelling in soil, dung, or rotten wood (Herting 1960; Arnaud 1978; Belshaw 1993).

The taxonomy of some *Dinera* species still needs to be clarified. One of the most problematic ones is *D. carinifrons* (Fallén). Ziegler and Lange (2001, 2007) pointed out that in the European Alps, two taxa, tentatively identified as *D. carinifrons* but probably corresponding to two different species, can be distinguished. Recently, *D. fuscata* Zhang and Shima was described from China and Japan (Zhang and Shima 2006), showing a close relationship to *D. carinifrons* and thus raising a need for a revision of the European material.

In our study, molecular sequence data from two mitochondrial genes (12S and 16S rDNA) were used to examine relationships among several Palaearctic *Dinera* spp. with the aim to solve the identities of *D. carinifrons* and *D. fuscata*. As the two morphotypes discussed by Ziegler and Lange (2001, 2007) differ, besides other characters, particularly in proportions of the head, in addition to molecular phylogenetics, morphometric methods were also applied to the same taxa and specimens. Choosing different methods for complementarity in taxonomic studies generally increases rigor in species delimitation and meets the principles of integrative (polyphasic) taxonomy (Schlick-Steiner et al. 2010; Muráriková et al. 2011; Yeates et al. 2011). A combination of molecular phylogenetic and morphometric analyses enables testing whether the specimens characterized by a shared morphological pattern are natural groups and testing or revealing morphological characters useful for the diagnosis and identification of these groups. Our study on a small, problematic group of *Dinera* may also provide a methodological example on a way to solve taxonomical problems in other taxa of the Tachinidae or other groups of Diptera, including those with economic importance, as many tachinids are natural enemies of insect agricultural and forest pests that are frequently used in biocontrol programs (Grenier 1988; Coombs and Sands 2000; Frank et al. 2006). A solid taxonomic knowledge of parasitoids is generally needed for effective biological control of their hosts (Smith et al. 2011).

## Materials and Methods

### Examination of material

The material examined (Table 1) was mostly dry and pinned. Some freshly collected specimens were preserved in ethanol for molecular analyses. The material came from the following institutions (names of curators in parentheses) and private collections:

Private collection of M. Barták, Prague, Czech Republic; Private collection of C. Bergström, Uppsala, Sweden; Biological Laboratory, Kyushu University, Fukuoka, Japan; (H. Shima); Private collection of E. Lutovinovas, Vilnius, Lithuania; Naturhistoriska Riksmuseet, Stockholm, Sweden (Y. Brodin); Institute of Entomology, Shenyang Normal University, Shenyang, China (C.-T. Zhang); Private collection of J. Vaňhara, Brno, Czech Republic; Museum für Naturkunde, Leibniz Institute for Research on Evolution and Biodiversity at the Humboldt University, Berlin, Germany (J. Ziegler).

The morphological terminology and definitions of many characters used in this paper were adopted from Tschorsnig and Herting (1994) and Merz and Haenni (2000).

*Dinera carinifrons* and *D. fuscata* are treated here together as the *D. carinifrons* species complex. This is defined here as follows: abdominal syntergite 1+2 excavated at most to 2/3 way to posterior margin; normally 3+3 dorsocentral setae present; costal seta undeveloped; relative length of second, third and fourth sections of costa approximately as 1:2:1 and wing cell R5 open; frontal vitta at least as wide as fronto-orbital plate at middle in both sexes. In this definition, *D. carinifrons* species complex includes only *D. carinifrons* and *D. fuscata*, whereas other Palaearctic species of *Dinera* seem to be more distantly related (Zhang and Shima 2006). The European material of *D. carinifrons* species complex has previously been identified and recorded as *D. carinifrons* in literature (Tschorsnig and Herting 1994; Cerretti 2010). Following Ziegler and Lange (2001, 2007), two morphotypes can be distinguished in European material, which we treated for the analyses as:

*D. carinifrons* A: corresponding to material from lowlands to moderate elevations of Europe and the Middle East, characterized by a tessellate greyish white microtrichosity, a slightly smaller body size, and particularly by the relatively narrower frons and parafacial

*D. carinifrons* B: corresponding to material from higher altitudes of Europe (predominantly the Alps), characterized by a dense yellowish grey microtrichosity, slightly larger body size, and particularly by the relatively broader frons and parafacial

Both morphotypes were represented in our material (Table 1). The specimens of *D. fuscata* from eastern Asia (China and Japan) were examined, and they were identified by C.-T. Zhang and H. Shima, including paratypes of this species. Zhang and Shima (2006) mentioned a variation in the colour of the palpus for *D. fuscata* that they treated as intraspecific. To test a possible taxonomic significance of this character, it was treated separately in some of our analyses:

*D. fuscata* A: corresponding to a form with a black or dark brown palpus, represented in the examined material by paratypes from the type locality in Japan and a few additional specimens from China

*D. fuscata* B: corresponding to a form with a pale (dark yellowish) palpus, represented in the examined material by a few specimens from China

Three additional species of the genus, *D. ferina* (Fallén), *D. grisescens* (Fallén), and *D. xuei* Zhang and Shima, were also included in both phylogenetic and morphometric analyses. *D. ferina* and *D. grisescens* are the only species of *Dinera* that occur sympatrically with *D. carinifrons* species complex in the western Palaearctic region. *D. xuei*, described from China, was regarded as being the most similar species to *D. carinifrons* and *D. fuscata* in morphology (Zhang and Shima 2006) and may thus represent a potential sister species to *D. carinifrons* and *D. fuscata*. One more species, *D. takanoi* (Mesnil, 1957), was contributed from GenBank (www.ncbi.nlm.nih.gov/genbank) and included in the phylogenetic analysis (the material of *D. takanoi* was not directly available for the morphometric analysis). Four outgroup taxa were added to root the resulting phylogenetic trees: *Billaea triangulifera* (Zetterstedt), (Diptera: Tachinidae: Dexiinae) *Dexia rustica* (Fabricius), *Dufouria chalybeata* (Meigen) and *Eriothrix rufomaculata* (De Geer).

Altogether 28 specimens were used for DNA sequencing and molecular phylogenetic analyses. The same specimens were also measured and included in the morphometric analyses. Morphometric data were further recorded for numerous additional specimens available from collections. Altogether 126 specimens (75 males, 51 females) were used for the morphometric part of the study, including a part of the type series of *D. carinifrons* (Table 1).

### Gene sequences analyses

Two mitochondrial markers, 12S and 16S rDNA, were analyzed (Table 2). The suitability of a combination of these two gene markers for reconstruction of phylogeny has been shown in different insect groups, including various families of Diptera (Flook and Rowell 1997; Skevington and Yeates 2000; Cook et al. 2004; Roháček et al. 2009). These markers have been also used with success for identification of cryptic females of the Tachinidae (Lutovinovas 2012).

Nucleic acids were extracted from mostly dried adults using DNeasy Blood and Tissue Kit (Qiagen, www.qiagen.com) following the manufacturer's protocol. Segments of the 12S and 16S rDNA were amplified using the primers 12Sma (5′ CTGGGATTAGATACCCTGTTAT) and 12Smb (5′ CAGAGAGTGACGGGCGATTTGT) (Cook et al. 2004), and the modified primers mt32 (5′ CAACATCGAGGTCGC) and mt34 (5′ TTGACCGTGCAAAGGTAG) (Nirmala et al. 2001). PCR products were visualized in 1% agarose gels, purified using the QIAquick PCR Purification Kit (Qiagen), and used directly for sequencing. The sequencing reactions were performed in a 10 μL reaction mixture using the Big Dye Terminator v. 3.1 chemistry (Applied Biosystems, Life Technologies, www.lifetechnologies.com). After the thermocycling, the reactions were purified with XTerminator® before injection into the ABI 3130 Genetic Analyzer (both from Applied Biosystems). More detailed protocols for the PCR amplifications may be consulted in Roháček et al. (2009).

Sequences were manually processed, and contigs were assembled using Sequencher v. 4.8 (GeneCodes, www.genecodes.com). Datasets were first examined for base comparison bias in MEGA v. 5 (Tamura et al. 2011). The computing of pairwise-distances was performed using PAUP* v. 4.0b10 (Swofford 2002) with GTR model criterion for distance correction.

To evaluate the best fit model for the Bayesian inference and maximum likelihood analyses, the combined dataset was partitioned into two gene regions (12S and 16S). Each of the partitions was evaluated in MrModeltest v. 2.2 (Nylander 2004) using both hierarchical likelihood ratio tests and Akaike information criterion. Bayesian inference was conducted on molecular dataset in MrBayes v. 3.1 (Huelsenbeck and Ronquist 2001). The reliability of the resulting tree topology was determined by 2,000,000 generations. Maximum likelihood analysis was processed in Garli v. 2.0 (Zwickl 2006). Two independent runs of 5,000,000 generations using the default automated stopping criterion were carried out. Nodal support was assessed using a nonparametric bootstrap with 100 replicates. The resulting tree was edited in TreeView (Page 1996), and the layout was prepared using Adobe Photoshop 8.0 (www.adobe.com).

### Morphometric analyses

In each specimen examined within the morphometric part of the study, 19 characters were defined as different linear distances on the head and wing were measured. The traditional morphometric approach was chosen for its relative simplicity of recording the characters and, particularly, for a possibility to interpret and use the results of the analyses in a straightforward fashion, i.e., to use some selected characters or their combinations directly for diagnoses and identifications of taxa. Traditional morphometric characters including absolute lengths and ratios are often applied in keys for Tachinidae (e.g., Tschorsnig and Herting 1994; Zhang and Shima 2006). This is particularly true for the characters on the head of *Dinera* species (Tschorsnig and Herting 1994; Ziegler and Lange 2001, 2007; Zhang and Shima 2006), while the wing venation characters are easy to measure and are often recorded in different taxonomic studies of Diptera (Houle et al. 2003; Vaňhara et al. 2007; Muráriková et al. 2011). Drawbacks of the traditional morphometrics are frequently a high correlation of some measurements and limited information on shape of the analyzed structures/specimens (Zelditch et al. 2004).

**Figure 1.**
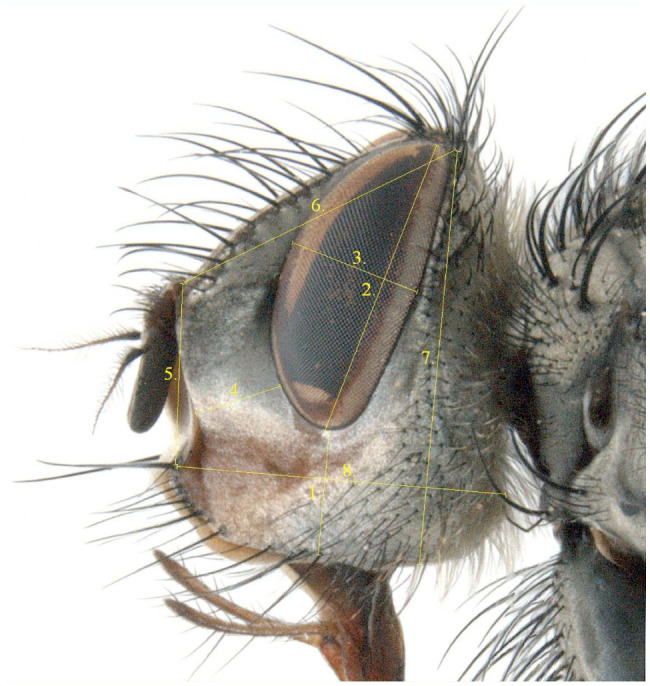
Left side of the head of *Dinera carinifrons* with delimitation of the characters measured for the morphometric analyses (code numbers of characters corresponding to Table 3). High quality figures are available online.

**Figure 2.**
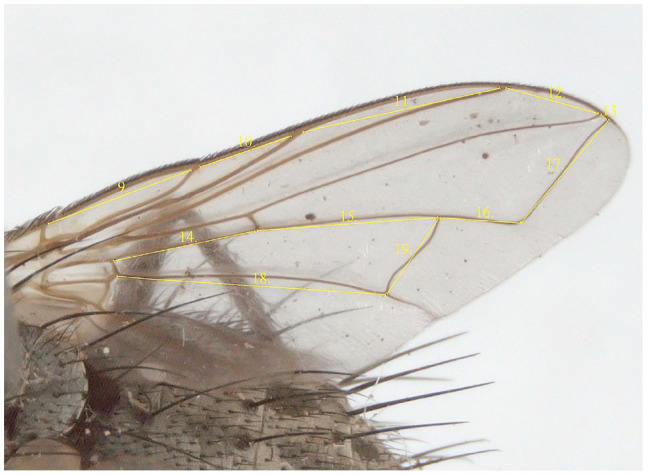
Right wing of *Dinera carinifrons* with delimitation of the characters measured for the morphometric analyses (code numbers of characters corresponding to Table 3). High quality figures are available online.

The list of the measured characters with their definitions is provided in Table 3; see also Figures 1–2 and Tschorsnig and Herting (1994). In all specimens under study, only the left side of the head (8 characters, focused in a plane perpendicular to exact lateral view) and the right wing (11 characters) were measured. Dry-mounted adult specimens were used, which were initially photographed using a stereomicroscope Olympus SZX 12 (www.olympus-global.com) with an attached Colour View IIIμ digital camera (one image for lateral view of head, one image for dorsal view of wing). The digitalized images were then scaled, and the characters were measured by means of the image analyzing software M.I.S QuickPhoto Micro (Promicra, www.promicra.com http://www.promicra.com).

To analyze the morphometric data, multivariate statistical methods were used (Tabachnick and Fidell 2006), which are suitable to examine multidimensional patterns of variation among morphological groups and have been often applied in taxonomy (e.g., Sorensen and Foottit 1992; Bustamante et al. 2004; Lozier et al. 2008). First, principal components analysis (PCA) based on a correlation matrix was carried out on the data set to determine the main components of variation in the morphometric data and to visualize the affinities among the examined specimens. This method does not assume any *a priori* grouping. Canonical discriminant analysis (CDA) was then used to test the differences among the groups (specified *a priori*) revealed by molecular methods/PCA and to determine those variables that contributed most to their separation. All statistical analyses were performed using Statistica v. 10 for Windows (Statsoft 2011).

## Results

### DNA sequences

**Sequence statistics**. Partial sequences of the mitochondrial genes 12S and 16S rDNA with total lengths of 361 and 364 bp, respectively, were obtained. Within these 725 sites in the resulting combined alignment, 19 positions contained a gap in one or more taxa, and 559 sites were constant (77.1 %). The alignment contained 99 parsimony-informative sites (13.7 %). The nucleotide composition of these genes showed a mean A+T content of 80.3% and 80.1% for the 12S and 16S, respectively. MrModeltest chose the model GTR + Г + I as favored for each of the individual gene regions.

**Figure 3.**
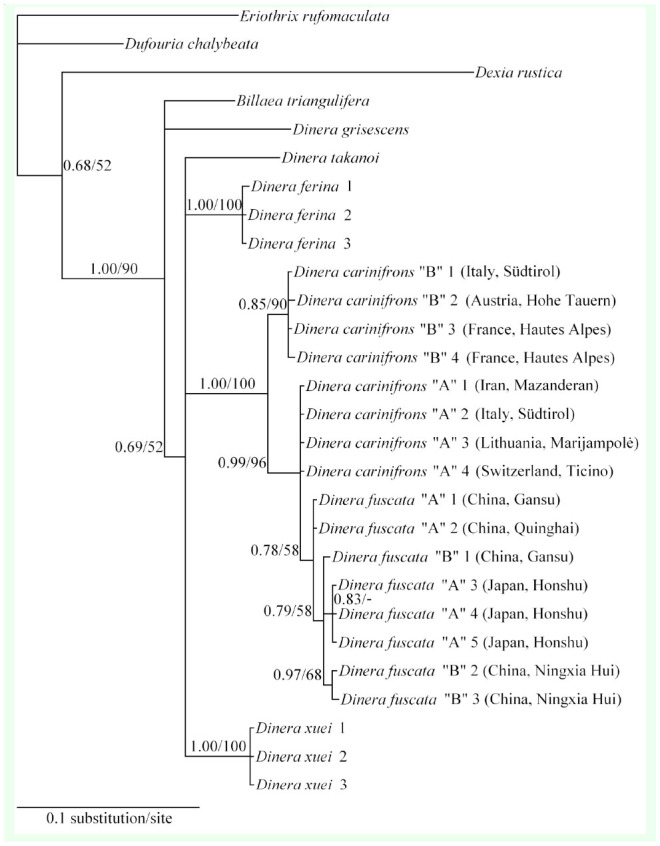
Phylogenetic reconstruction (topology based on Bayesian inference) of the combined 12S+16S rDNA dataset, showing Bayesian influence posterior probabilities and maximum likelihood bootstrap values above the branches. High quality figures are available online.

**Molecular phylogeny**. Both Bayesian inference and maximum likelihood analyses resulted in phylograms with the same topology from the combined dataset of 12S and 16S rDNA (Figure 3; the maximum likelihood tree differred from the Bayesian inference tree only in showing no support for a clade of *D. fuscata* specimens A3+A4+A5 from Honshu, Japan). A close relationship of *Billaea* to *Dinera* was confirmed. The *D. carinifrons* species complex was found to be monophyletic (posterior probability = 1.00; maximum likelihood bootstrap value = 100) with *D. carinifrons* B forming a sister clade to a well-supported group including *D. carinifrons* A, *D. fuscata* A, and *D. fuscata* B. Neither *D. fuscata* A nor *D. fuscata* B alone were demonstrated to be monophyletic, but there was some support from the analysis (posterior probability = 0.78; maximum likelihood = 58) for the monophyly of the two forms of *D. fuscata* together. The specimens analyzed of *D. ferina*, *D. xuei*, *D. carinifrons* B, and *D. carinifrons* A represented a single haplotype for each taxon. The intra-group genetic divergences within different samples of *D. fuscata* ranged from 0 to 0.58% for 12S rDNA and from 0 to 1.17% for 16S rDNA. The differences in the gene sequences between *D. carinifrons* A and different samples of *D. fuscata* were lower than 1% for 12S rDNA and lower than 1.6% for 16S rDNA. This was less than interspecific divergences between the other, mostly well-delimited Palaearctic *Dinera* spp. included in the study (12S rDNA: minimum 1.43 % between *D. carinifrons* A and B, maximum 9.46% between *D. grisescens* and *D. fuscata*; 16S rDNA: minimum 2.80% between *D. carinifrons* B and *D. fuscata*, maximum 11.92 % between *D. grisescens* and *D. fuscata*; Table 4).

**Figure 4.**
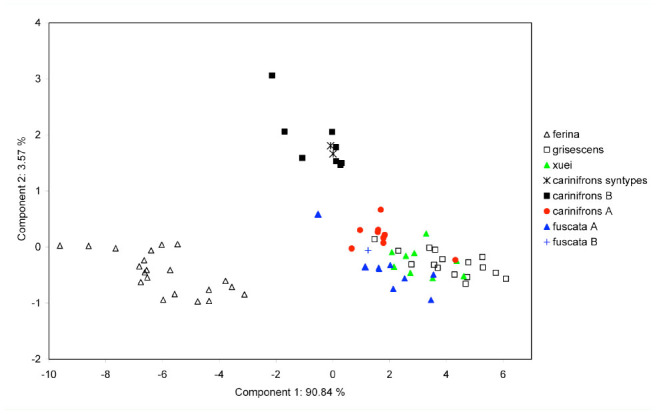
Principal component ordination of male specimens of *Dinera* spp. onto the first and second principal axes. High quality figures are available online.

### Morphometric data

**Figure 5.**
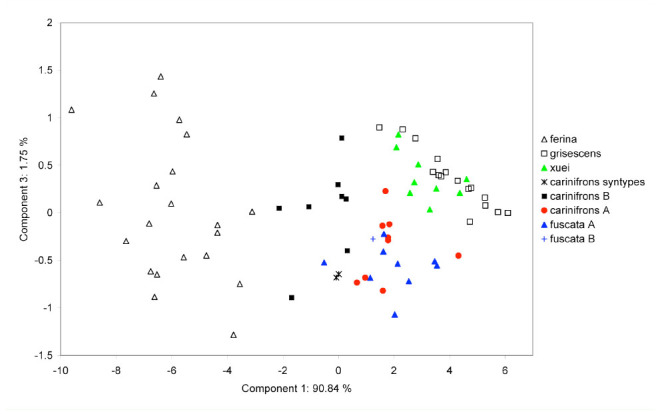
Principal component ordination of male specimens of *Dinera* spp. onto the first and third principal axes. High quality figures are available online.

**Figure 6.**
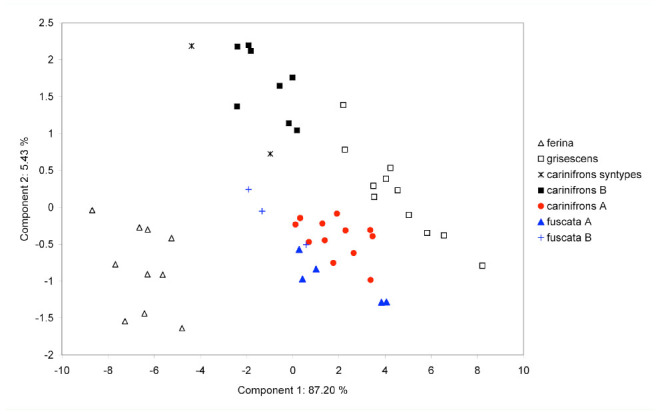
Principal component ordination of female specimens of *Dinera* spp. onto the first and second principal axes. High quality figures are available online.

Summary statistics of the measured characters for the males and females of all examined *Dinera* spp. are given in Tables 5–6. In the first step, the entire morphometric data set, including all examined *Dinera* spp., was analyzed with PCA. Males and females were analyzed separately to exclude the effect of sexual dimorphism. The PCA revealed similar patterns for both sexes. The projection of male specimens on the first two principal component axes is shown in Figure 4. *Dinera ferina* was largely separated from all other species along the first component axis, while *D. carinifrons* B occupied a space distinct from all other specimens, mainly along the second component axis. The distribution of *D. carinifrons* A, *D. fuscata* A and B, *D. grisescens*, and *D. xuei* partly overlapped in this projection. *Dinera grisescens* and *D. xuei* could be separated from *D. carinifrons* A and *D. fuscata* along the third component axis (Figure 5). The contributions of all measured characters to the components 1–3 (factor loadings) are given in Table 7. The first component was strongly correlated with most characters, suggesting that it represented mainly differences in general size. Specimens of *D. ferina* were distinctly larger than all remaining species. The separation of *D. carinifrons* B from all other taxa was mainly due to a larger parafacial width, which was the only variable having a relatively high factor loading with this axis. The third component (accounting, however, for only 1.7 % of the variation in the whole dataset) was correlated mainly with the length of the costal section 5 on the wing that was reduced in many specimens of *D. grisescens* and *D. xuei* and developed in specimens of the *D. carinifrons* species complex. The analysis of females gave similar results (Figure 6, Table 7), particularly in the relative contributions of characters and the groups of *D. ferina* and *D. carinifrons* B specimens being distinct from the remaining taxa. The projections of *D. carinifrons* A, *D. fuscata*, and *D. grisescens* hardly overlapped, unlike in males (females of *D. xuei* were, however, not available for the study).

**Figure 7.**
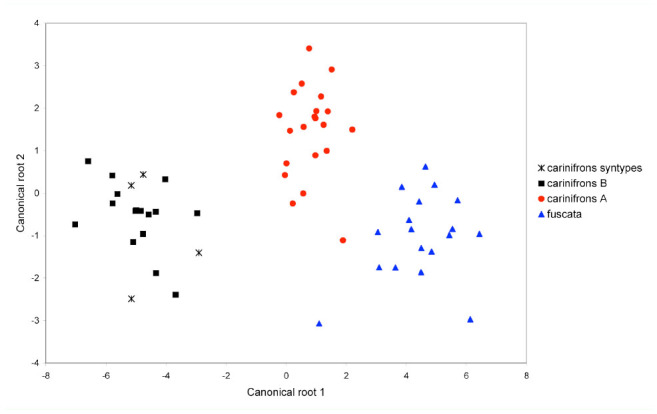
Scatterplot of canonical scores of specimens of *Dinera carinifrons* species complex (males and females) onto the first and second discriminant functions (canonical roots) resulting from canonical discriminant analysis. High quality figures are available online.

In the next step, CDA was used to analyze the *D. carinifrons* species complex in detail. As it is generally recommended for CDA to have a relatively high number of cases (specimens) in individual tested groups, both sexes were analyzed together and *D. fuscata* A and B were treated as a single group. This was also partly supported by the results of the molecular phylogenetic analysis and PCA. Three groups were thus *a priori* defined for CDA: *D. carinifrons* A and B and *D. fuscata*. The scatterplot of canonical scores of *D. carinifrons* complex specimens resulting from CDA is shown in Figure 7. In the plane defined by the two discriminant functions (canonical roots), all three groups could be wellseparated. The first discriminant function mainly separated *D. carinifrons* B from both *D. carinifrons* A and *D. fuscata*, and partly also the two latter groups from each other. The character with the greatest discriminatory power was clearly the parafacial width, which was the most correlated measure with the first discriminant function (Table 8). The second discriminant function enabled a partial separation of *D. carinifrons* A from *D. carinifrons* B and *D. fuscata*. The most correlated corresponding characters were lengths of certain wing vein sections, particularly Cs4. Based on a relatively low eigenvalue of the second discriminant function, the magnitude of the discrimination was, however, smaller (Table 8).

## Discussion and Conclusions

### Phylogenetic relationships and delimitation of taxa

The taxon sampling in this study limits conclusions on the monophyletic status of *Dinera*, which needs to be tested by inclusion of additional species from the genus as well as other closely related taxa. Further studies should concentrate on the delimitation of *Dinera* in respect to *Billaea*, as already pointed out by Zhang and Shima (2006) and Zhang and Fu (2012), and possibly also on other Dexiini taxa, especially on several Neotropical, Afrotropical, and Australian genera, which are currently poorly defined in respect to *Dinera* and *Billaea*. A broader taxon sampling that includes additional species of *Dinera* from eastern Palaearctic, Oriental, and Afrotropical Regions would also help to assess sister-group relationships within the genus.

Molecular and morphometric data clearly supported the distinctiveness of specimens from the Alps, provisionally named as *D. carinifrons* B in this paper. This taxon was found to be monophyletic in the molecular analysis as a sister-group to *D. carinifrons* A and *D. fuscata* together*.* Morphologically, *D. carinifrons* B can be differentiated from *D. carinifrons* A and *D. fuscata* by a slightly larger general size, a dense microtrichosity, and different head proportions, such as a larger parafacial width (0.43–0.59 mm in *D. carinifrons* B compared to 0.22–0.38 mm in *D. carinifrons* A and 0.19–0.42 mm in *D. fuscata* see also Tables 5 and 6). The opinion of Ziegler and Lange (2001, 2007) that the *D. carinifrons* species complex includes two different species in Europe is thus supported. The other European taxon, *D. carinifrons* A, is very close to eastern Palaearctic specimens of *D. fuscata* both in terms of molecular sequences and morphometric data. The genetic pairwise distances between the corresponding samples were equal to or lower than 1% in the combined 12S and 16S rDNA data. Although interspecific genetic distances in mtDNA may be relatively low in some taxa of the Tachinidae, even between morphologically welldiagnosable species, which may be due to a recent radiation of this group (Novotná et al. 2009), these distances between *D. carinifrons* A and *D. fuscata* were lower compared to the interspecific distances between well-defined *Dinera* species examined here (Table 4). Every individual morphometric character between *D. carinifrons* A and *D. fuscata* largely overlapped, although CDA was able to discriminate both groups of specimens based on their combination (Figure 7). A detailed morphological analysis of both groups further suggested a difference between western and eastern Palaearctic specimens in the relative length of the palpus, a character that was not included in the morphometric analysis as it was not possible to exactly measure it in all specimens examined (the palpus is frequently hidden in dry-mounted specimens). Further studies including additional material from over the distribution range and desirably also information on the biology and the hosts are needed to assess the taxonomic value of the molecular and morphometric differences between those western and eastern Palaearctic populations. The variation solely in the color of the palpus in the eastern Palaearctic *D. fuscata* specimens (corresponding to specimens denoted as *D. fuscata* A and B in this study) is treated as intraspecific in concordance with Zhang and Shima (2006).

### Nomenclature and taxonomy

The examination of the type series of *Musca carinifrons* Fallén deposited in Naturhistoriska Riksmuseet by J. Ziegler revealed that it consisted of a mix of specimens that can be assigned to both *D. carinifrons* A and B. All four syntypes (two males, two females) of *M. carinifrons* that were included in the morphometric analyses in our study were convincingly classified by both PCA and CDA as belonging to the same group as *D. carinifrons* B (Figures 4–7; see also Tables 5 and 6). According to Article 74 of ICZN (1999), we designate here one of these specimens as the lectotype for *M. carinifrons* to stabilize the nomenclature in the group. The lectotype is deposited in Naturhistoriska Riksmuseet. It is a dry-mounted (pinned) male with an original label handwritten on white paper “M. cari- / nifrons ♂.“ J. Ziegler added a further label printed on white paper, “Dinera ♂ / carinifrons / (Fallén, 1817) / det. J. Ziegler 2012,“ and a red label with the printed data “LECTOTYPUS / Musca / carinifrons ♂ / Fallén, 1817 / des. J. Ziegler, 2012.“ The lectotype is well preserved. Only the left mid-leg and the left antenna are missing.

A further syntype female with a white original label had the following handwritten data: “M. cari- / nifrons ♀.“ One male and seven further females without labels were labelled as “Dinera / carinifrons / (Fallén, 1817) / det. J. Ziegler 2012“ (printed on white paper). Another one male and two females without labels were labelled as “Dinera / fuscata / ZHANG &SHIMA 2006 / det. J. Ziegler 2012.“ All these former syntypes apart from the lectotype (two males, nine females) were labelled additionally with red labels and the printed data “PARALECTOTYPUS / Musca / carinifrons / Fallén, 1817 / des. J. Ziegler, 2012.”

A male of *Dinera fuscata* with an original label handwritten on pale green paper “M. autum- / nalis ♂/ mihi“ and a female with an original label “98“ have been excluded from the type series of *M. carinifrons*.

The lectotype is thus considered to be conspecific with *D. carinifrons* B in this study. This interpretation of *D. carinifrons* based on the present lectotype designation is in accordance with the interpretation of *D. carinifrons* by Zhang and Shima (2006). Most of the specimens morphologically corresponding to *D. carinifrons* examined in this study were collected by J. Ziegler in the Alps, but some specimens from older collections from Germany, Scandinavia, Siberia, and the Russian Far East were also examined. This suggests that *D. carinifrons*, as redefined here, has a wider, transpalaearctic distribution that will be reviewed in detail in a separate paper.

Pending a more detailed study in future, the western Palaearctic *D. carinifrons* A and eastern Palaearctic *D. fuscata* are interpreted here as geographical forms of one species, *Dinera fuscata* Zhang and Shima. We do not formally assign a name to the western Palaearctic form according to the ICZN here.

The characters of the vittae on the scutum and the number of acrostichal setae mentioned as diagnostic characters between *D. carinifrons* and *D. fuscata* by Zhang and Shima (2006) are variable and not reliable for distinguishing the species. Based on our study and the present lectotype designation, a revised differential diagnosis for *D. carinifrons* and *D. fuscata* is provided below that would replace the couplet no. 17 in the determination key to Palaearctic *Dinera* by Zhang and Shima (2006) as follows:

17. Larger, grey species; body length 6.5–10.8 mm; abdomen with dense yellowish-grey (rarely bluish-grey) microtrichosity with only a light tessellate appearance, but when seen from a very low angle from behind the microtrichosity is dense and covers the whole of the abdominal tergites including abdominal dorsum. Male: Frons at its narrowest point 0.26–0.42 times as wide as eye in dorsal view; parafacial wide, in profile at its narrowest point 0.5–0.8 times as wide as the horizontal width of eye; postabdomen elongated, syncercus flat in lateral view, paramere slightly longer than basiphallus. Female: Frons at its narrowest point 1.05–1.35 times as wide as eye in dorsal view; medial (inner) vertical setae about 0.75–0.95 of eye height. _ _ _ _ _ *D. carinifrons* (Fallén)

- Smaller, dark species; body length 5.3–9.9 mm; abdomen with a tessellate appearance, also when seen from a very low angle from behind, with sparse greyish-white microtrichosity laterally and dark brownish dorsally. Male: Frons at its narrowest point 0.15–0.30 times as wide as eye in dorsal view; parafacial narrow, in profile at its narrowest point 0.35– 0.62 times as wide as the horizontal width of eye; postabdomen with a short syncercus, convex in lateral view, paramere slightly shorter than basiphallus. Female: Frons at its narrowest point 0.95–1.25 times as wide as eye in dorsal view; medial (inner) vertical setae about 0.60–0.85 of eye height. _ _ _ _ _ *D. fuscata* Zhang & Shima

**Table 1.**
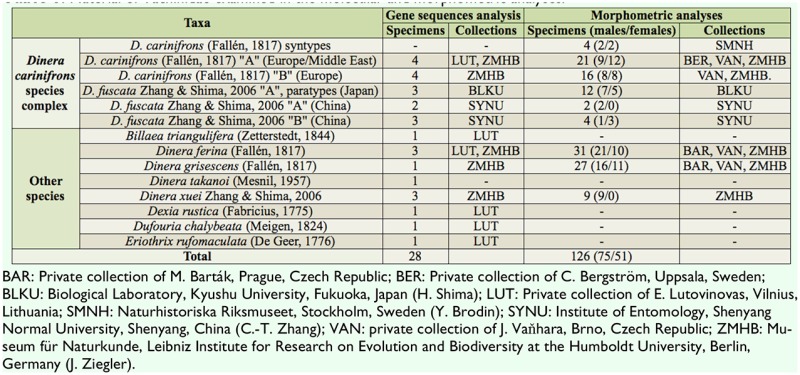
Material of Tachinidae examined in the molecular and morphometric analyses.

**Table 2.**
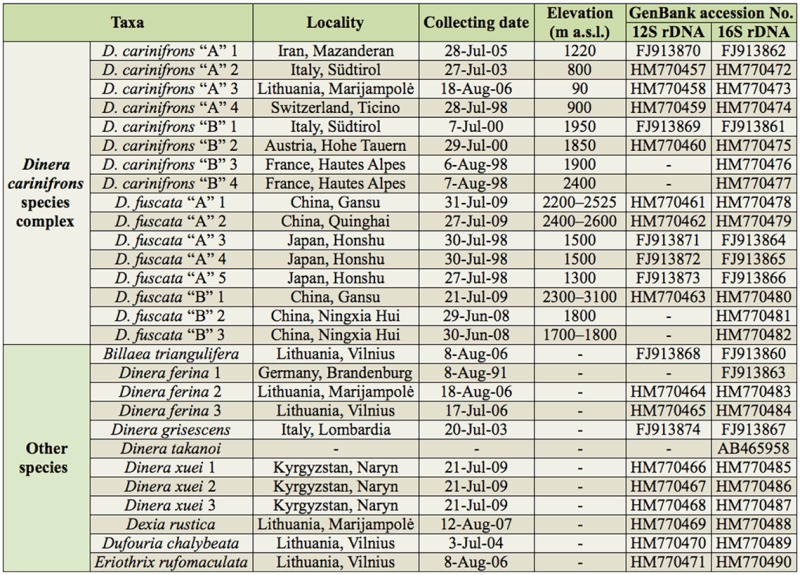
GenBank accession numbers for specimens in gene sequences analysis.

**Table 3.**
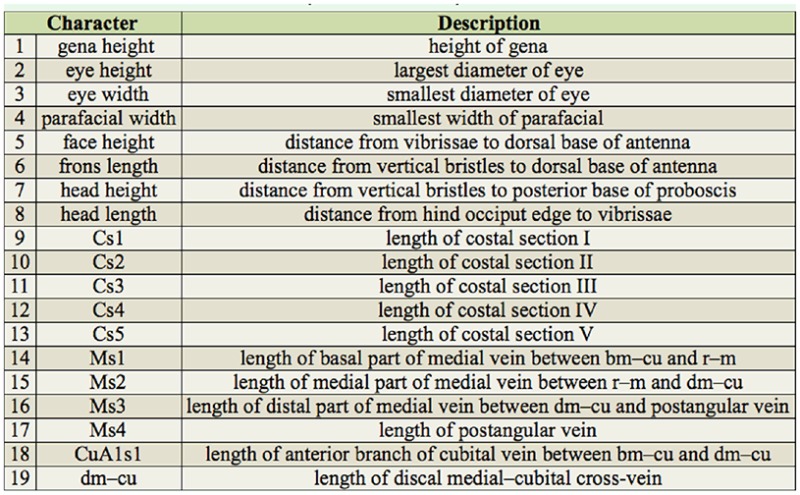
List of the characters measured in the morphometric analyses.

**Table 4.**
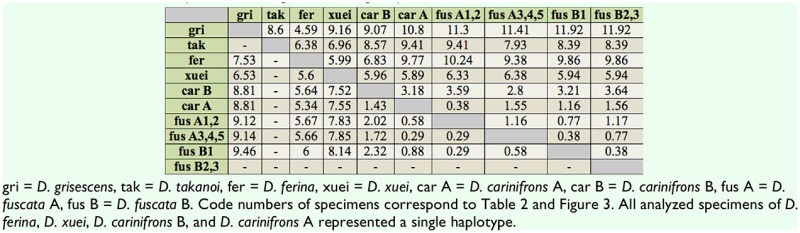
Pairwise distances, converted into percents, between samples of the Palaearctic *Dinera*, obtained from the analysis of the mitochondrial rDNA (12S left, 16S right of the diagonal).

**Table 5.**
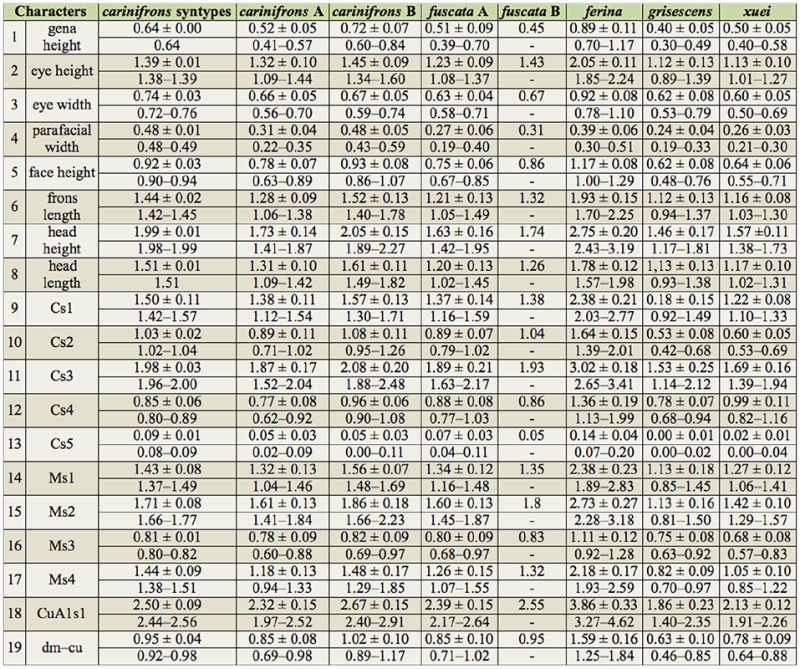
Summary statistics for the morphometric characters measured in males of *Dinera* species/forms (mean ± standard deviation; minimum–maximum; all values in mm).

**Table 6.**
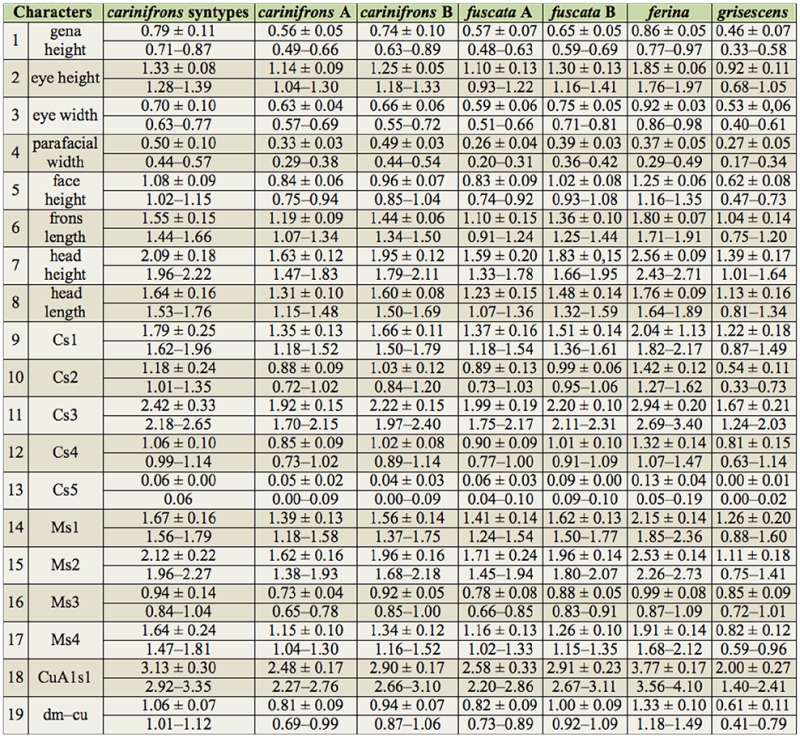
Summary statistics for the morphometric characters measured in females of *Dinera* species/forms (mean ± standard deviation; minimum-maximum; all values in mm).

**Table 7.**
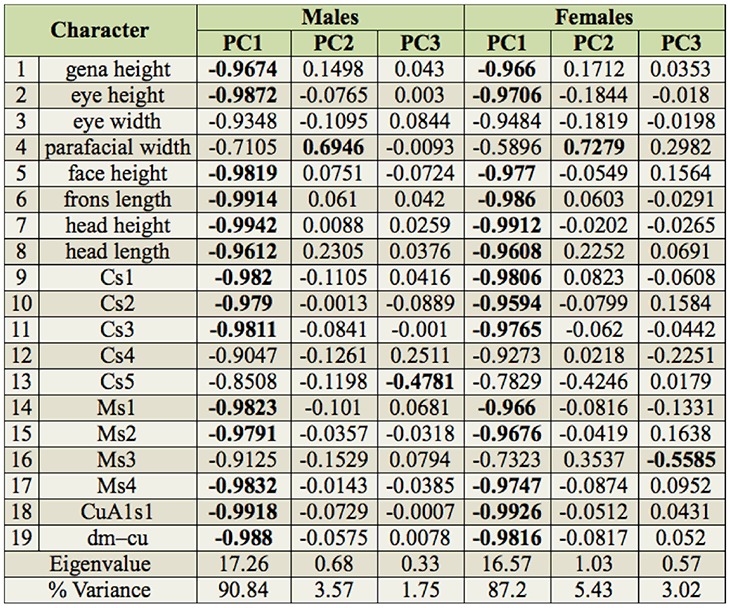
Correlations (factor loadings) of morphometric characters with principal component axes 1–3 in PCA of *Dinera* spp. (highest values in bold).

**Table 8.**
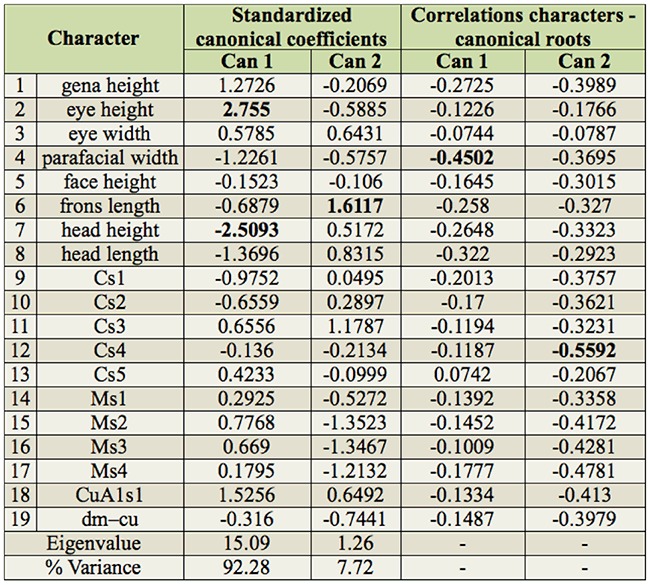
Standardized canonical coefficients and correlations of characters with two discriminant functions (canonical roots) resulting from canonical discriminant analysis of *Dinera carinifrons* species complex (highest values in bold).

## Abbreviations

CDA,: canonical discriminant analysis;
**PCA**,: principal component analysis
